# Genome sequence for the thick topshell, *Phorcus lineatus *(da Costa, 1778)

**DOI:** 10.12688/wellcomeopenres.19485.1

**Published:** 2023-07-11

**Authors:** Nova Mieszkowska, Rob Mrowicki, Marine Biological Association Genome Acquisition Lab, Darwin Tree of Life Barcoding collective, Wellcome Sanger Institute Tree of Life programme, Wellcome Sanger Institute Scientific Operations: DNA Pipelines collective, Tree of Life Core Informatics collective, Darwin Tree of Life Consortium

**Affiliations:** 1 The Marine Biological Association, Plymouth, England, UK

**Keywords:** Phorcus lineatus, thick topshell, genome sequence, chromosomal, Trochidae

## Abstract

We present a genome assembly from an individual *Phorcus lineatus *(the thick topshell; Mollusca; Gastropoda; Trochida; Trochidae). The genome sequence is 958 megabases in span. Most of the assembly (99.9%) is scaffolded into 18 chromosomal pseudomolecules. The mitochondrial genome has also been assembled and is 19.1 kilobases in length.

## Species taxonomy

Eukaryota; Metazoa; Spiralia; Lophotrochozoa; Mollusca; Gastropoda; Vetigastropoda; Trochida; Trochoidea; Trochidae; Cantharidinae; *Phorcus*; *Phorcus lineatus* (da Costa, 1778) (NCBI:txid1620919).

## Background

The prosobranch gastropod *Phorcus lineatus* (da Costa, 1778), commonly called the thick topshell or toothed topshell, is a species of trochid commonly found on moderately exposed rocky shores in the south and west of the UK. It has a geographic distribution extending from Morocco ( Lewis, 1996) to north Wales ( Mieszkowska & Sugden, 2022). *P. lineatus* occurs among boulders, cobbles, and bedrock in the high and midshore zones of rocky intertidal habitats. Some seasonal migration downshore in the winter has been previously recorded ( Crothers, 2001), but with climate change this has not been evident in UK populations studied between the 2000s and 2020s (MarClim unpublished data).

*P. lineatus* is one of the most abundant species of grazer occurring on rocky shores in the north-east Atlantic, and feeds off biofilm on rock surfaces ( Desai, 1966). *P. lineatus* is a broadcast spawner with a lecithotrophic larval stage lasting for a few days ( Desai, 1966). Recruits settle within the same intertidal habitat as the adults, but in cryptic habitats ( Crothers, 2001) under boulders, cobbles, and in crevices. Maturation occurs at around two years of age ( Garwood & Kendall, 1985), and individuals can live in excess of 20 years ( Mieszkowska *et al.*, 2006). *P. lineatus* is distinguished from other species of trochid by its black conical shell, often with an eroded apex exposing the nacreous shell layer. Growth checks are evident as vertical bands around the shell. There is a white columella projection near to the aperture which explains its common name.

This species has shown some of the fastest responses to climate change in any natural system, expanding the leading range edge in north Wales over 10 km per decade ( Mieszkowska *et al.*, 2006; Mieszkowska & Sugden, 2016). A high-quality genome sequence for this species will allow future studies to understand more about the mechanisms driving the observed response of this species to a changing climate.

## Genome sequence report

**Figure 1.**
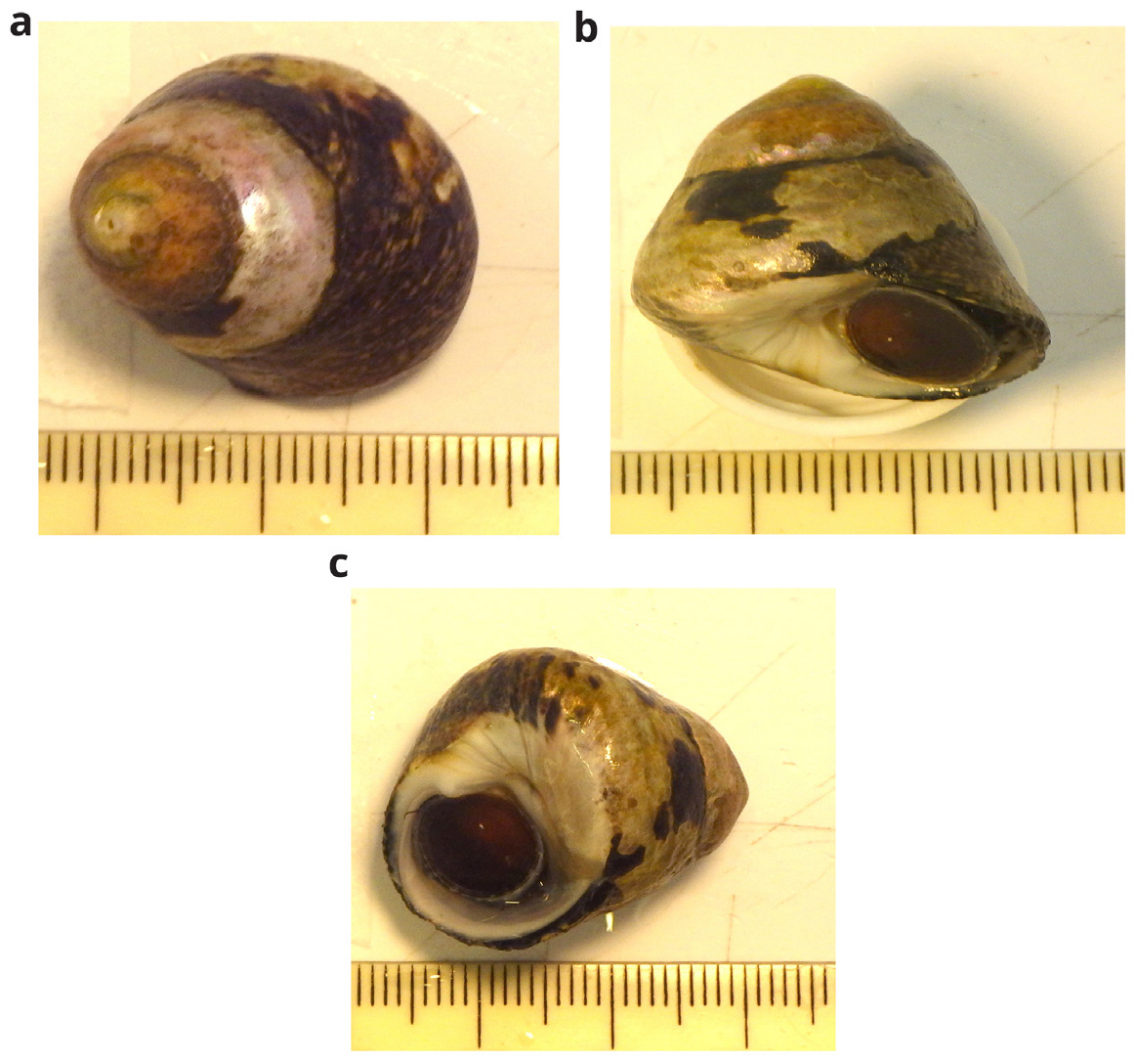
Photographs of the *Phorcus lineatus* (xgPhoLine1) specimen used for genome sequencing.

The genome was sequenced from one *Phorcus lineatus* ( Figure 1) collected from Godrevy in Cornwall, UK (latitude 50.24, longitude –5.396). A total of 32-fold coverage in Pacific Biosciences single-molecule HiFi long reads and 51-fold coverage in 10X Genomics read clouds were generated. Primary assembly contigs were scaffolded with chromosome conformation Hi-C data. Manual assembly curation corrected 225 missing joins or misjoins and removed 83 haplotypic duplications, reducing the assembly length by 3.87% and the scaffold number by 67.9% and increasing the scaffold N50 by 3.44%.

**Figure 2.**
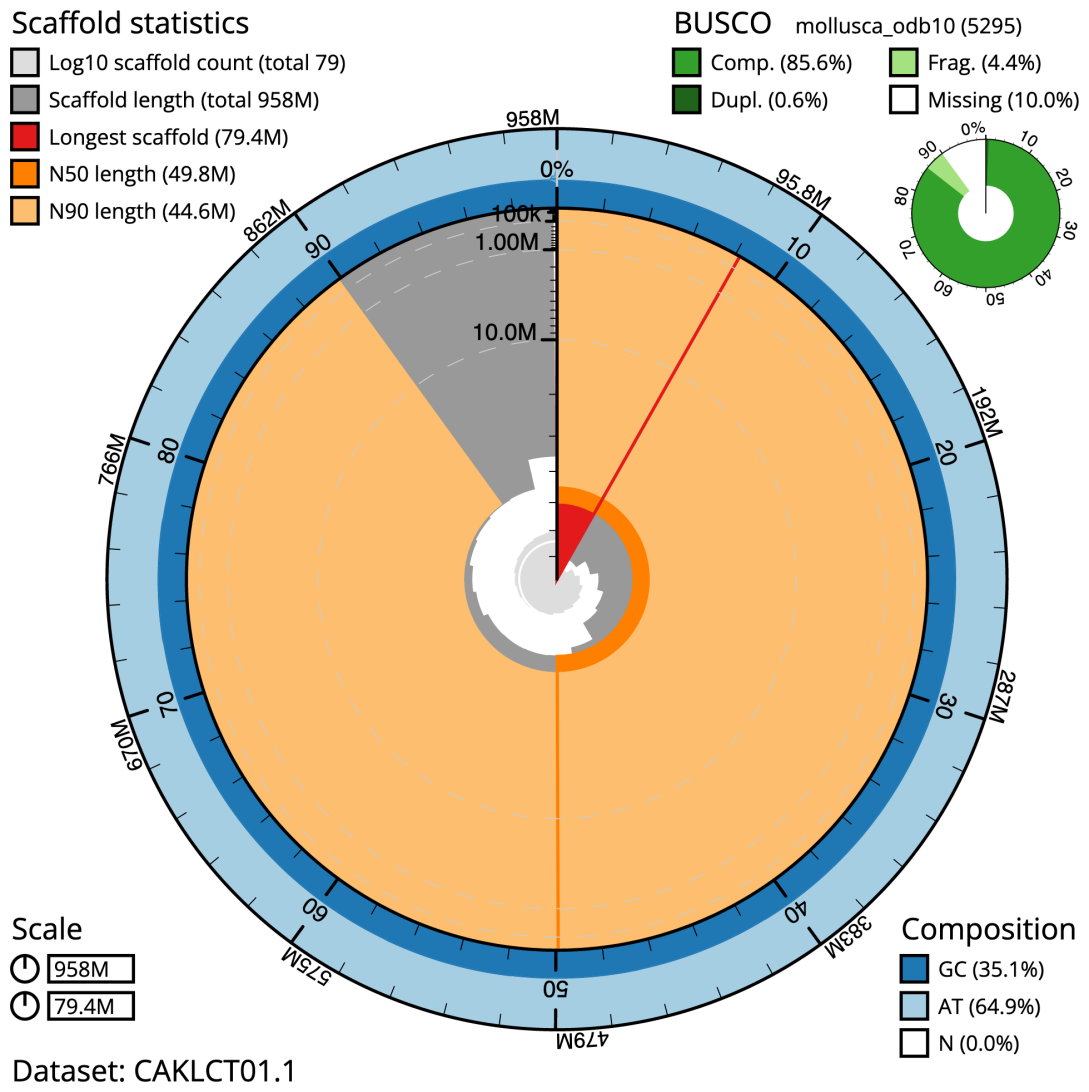
Genome assembly of *Phorcus lineatus*, xgPhoLine1.1: metrics. The BlobToolKit Snailplot shows N50 metrics and BUSCO gene completeness. The main plot is divided into 1,000 size-ordered bins around the circumference with each bin representing 0.1% of the 957,853,961 bp assembly. The distribution of scaffold lengths is shown in dark grey with the plot radius scaled to the longest scaffold present in the assembly (79,430,111 bp, shown in red). Orange and pale-orange arcs show the N50 and N90 scaffold lengths (49,790,511 and 44,639,602 bp), respectively. The pale grey spiral shows the cumulative scaffold count on a log scale with white scale lines showing successive orders of magnitude. The blue and pale-blue area around the outside of the plot shows the distribution of GC, AT and N percentages in the same bins as the inner plot. A summary of complete, fragmented, duplicated and missing BUSCO genes in the mollusca_odb10 set is shown in the top right. An interactive version of this figure is available at https://blobtoolkit.genomehubs.org/view/xgPhoLine1.1/dataset/CAKLCT01.1/snail.

The final assembly has a total length of 958 Mb in 78 sequence scaffolds with a scaffold N50 of 49.8 Mb ( Table 1). Most (99.9%) of the assembly sequence was assigned to 18 chromosomal-level scaffolds ( Figure 2– Figure 5; Table 2). Chromosome 13 contains a possible heterozygous inversion in the region of approximately 1–6.14 Mb. The orientation of the chromosome between these coordinates is not certain.

**Table 1.** Genome data for *Phorcus lineatus*, xgPhoLine1.1.

Project accession data		
Assembly identifier	xgPhoLine1.1	
Species	*Phorcus lineatus*	
Specimen	xgPhoLine1	
NCBI taxonomy ID	1620919	
BioProject	PRJEB48398	
BioSample ID	SAMEA7536116	
Isolate information	xgPhoLine1, muscle tissue (DNA sequencing and Hi-C scaffolding) xgPhoLine2, muscle tissue (RNA sequencing)	
Assembly metrics *		*Benchmark*
Consensus quality (QV)	56.5	*≥ 50*
*k*-mer completeness	99.99%	*≥ 95%*
BUSCO **	C:85.6%[S:85.0%,D:0.6%], F:4.4%,M:10.0%,n:5,295	*C ≥ 95%*
Percentage of assembly mapped to chromosomes	99.9%	*≥ 95%*
Sex chromosomes	-	*localised homologous pairs*
Organelles	Mitochondrial genome assembled	*complete single alleles*
Raw data accessions		
PacificBiosciences SEQUEL II	ERR7254638, ERR7254639	
10X Genomics Illumina	ERR7220469–ERR7220472	
Hi-C Illumina	ERR7220468, ERR7220473–ERR7220475	
PolyA RNA-Seq Illumina	ERR10377999	
Genome assembly		
Assembly accession	GCA_921293015.1	
*Accession of alternate haplotype*	GCA_921293055.1	
Span (Mb)	957.8	
Number of contigs	428	
Contig N50 length (Mb)	4.9	
Number of scaffolds	78	
Scaffold N50 length (Mb)	49.8	
Longest scaffold (Mb)	79.4	

* Assembly metric benchmarks are adapted from column VGP-2020 of “Table 1: Proposed standards and metrics for defining genome assembly quality” from ( Rhie *et al.*, 2021).

** BUSCO scores based on the mollusca_odb10 BUSCO set using v5.3.2. C= complete [S= single copy, D=duplicated], F=fragmented, M=missing, n=number of orthologues in comparison. A full set of BUSCO scores is available at https://blobtoolkit.genomehubs.org/view/xgPhoLine1.1/dataset/CAKLCT01.1/busco.

**Figure 3.**
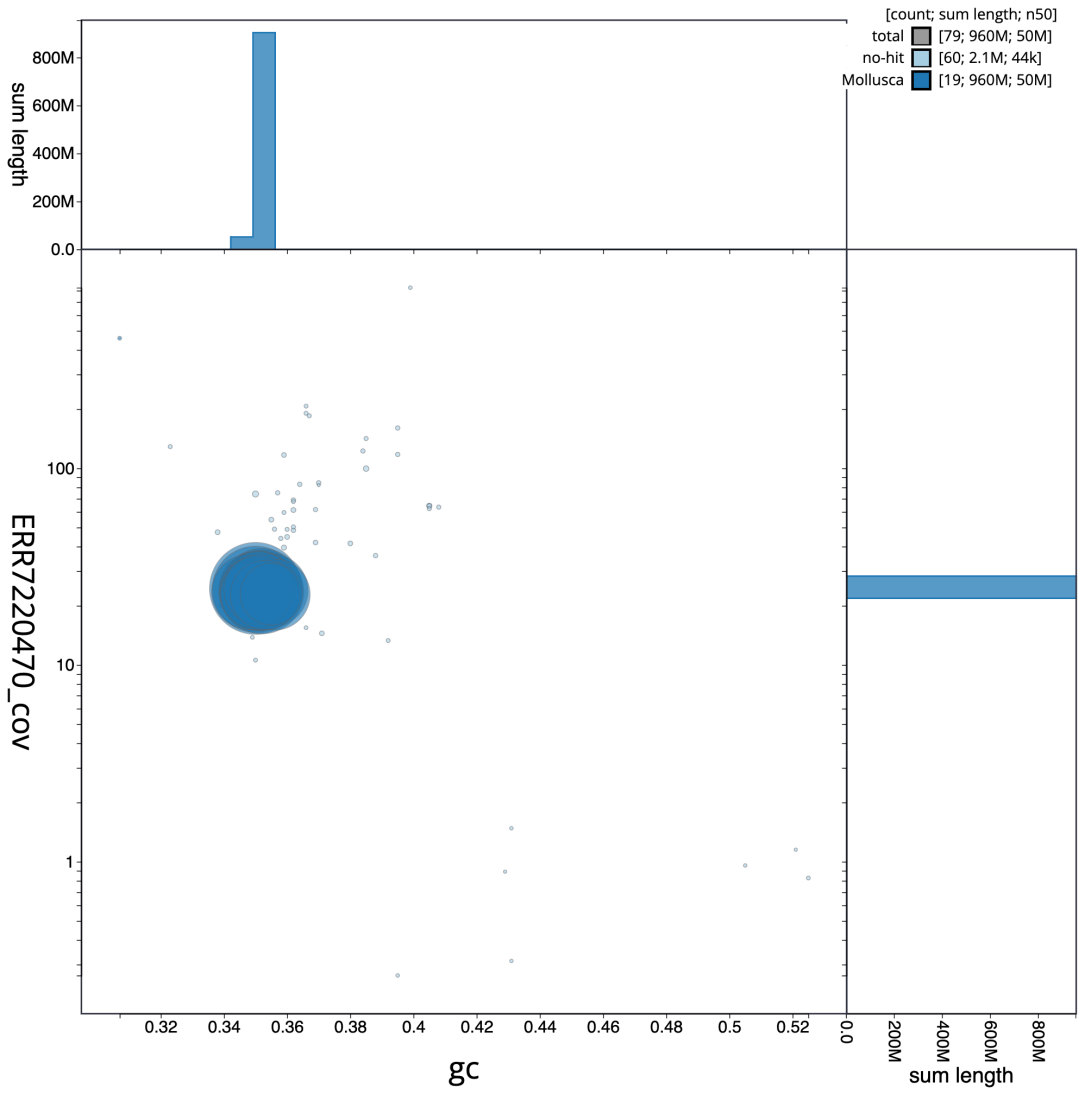
Genome assembly of *Phorcus lineatus*, xgPhoLine1.1: GC coverage. BlobToolKit GC-coverage plot. An interactive version of this figure is available at https://blobtoolkit.genomehubs.org/view/xgPhoLine1.1/dataset/CAKLCT01.1/blob.

**Figure 4.**
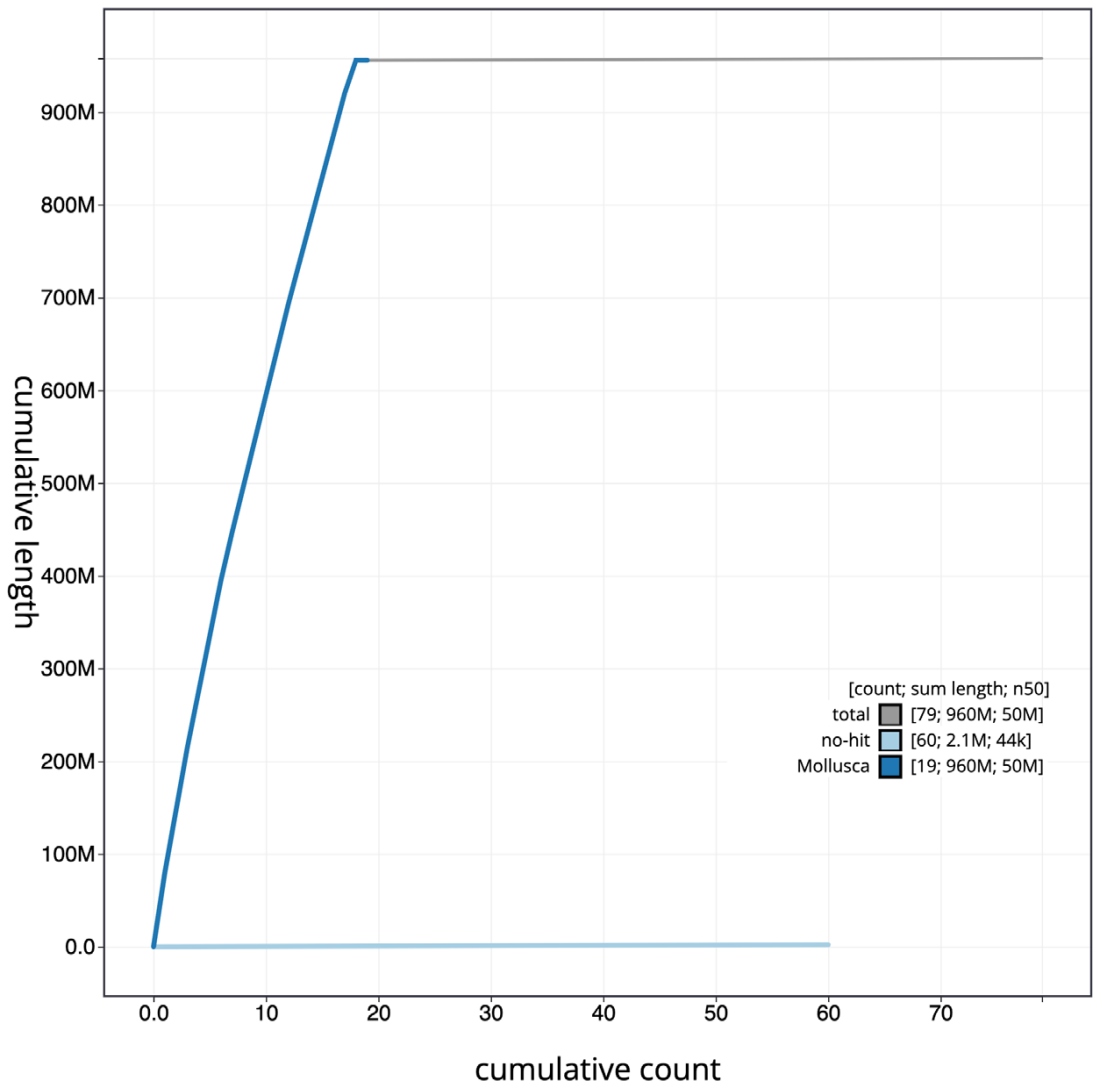
Genome assembly of *Phorcus lineatus*, xgPhoLine1.1: cumulative sequence. BlobToolKit cumulative sequence plot. An interactive version of this figure is available at https://blobtoolkit.genomehubs.org/view/xgPhoLine1.1/dataset/CAKLCT01.1/cumulative.

**Figure 5.**
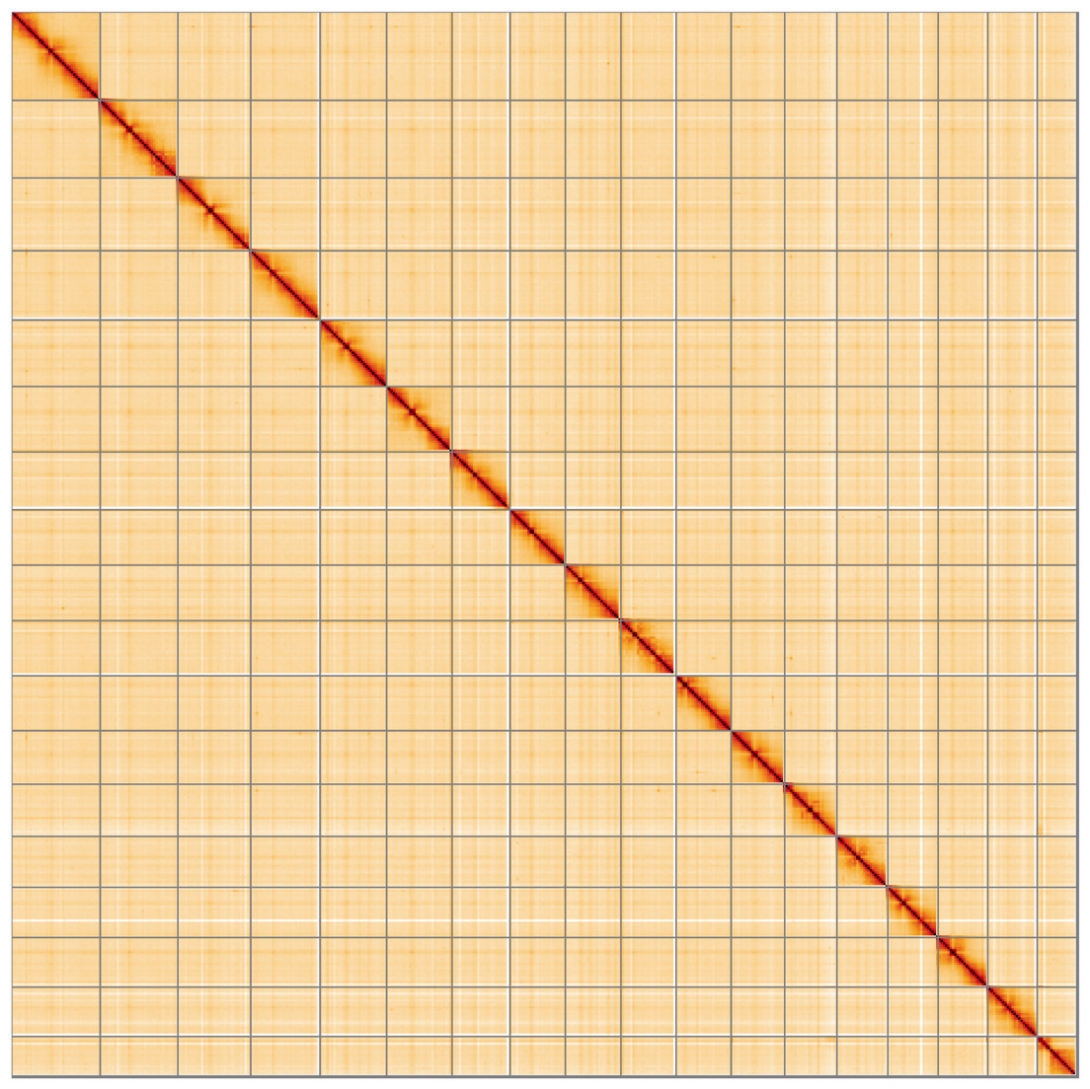
Genome assembly of *Phorcus lineatus*, xgPhoLine1.1: Hi-C contact map. Hi-C contact map of the xgPhoLine1.1 assembly, visualised using HiGlass. Chromosomes are given in order of size from left to right and top to bottom. An interactive version of the figure can be viewed at https://genome-note-higlass.tol.sanger.ac.uk/l/?d=V15PjfA4QSC1MB2tiWmxzA.

**Table 2.** Chromosomal pseudomolecules in the genome assembly of *Phorcus lineatus*, xgPhoLine1.

INSDC accession	Chromosome	Size (Mb)	GC%
OV121081.1	1	79.43	35
OV121082.1	2	69.66	35
OV121083.1	3	65.39	35.2
OV121084.1	4	62.25	35.2
OV121085.1	5	59.84	35.1
OV121086.1	6	58.7	35.1
OV121087.1	7	52.04	34.8
OV121088.1	8	49.79	35.1
OV121089.1	9	49.65	35.5
OV121090.1	10	49.55	35.2
OV121091.1	11	49.44	35.1
OV121092.1	12	47.87	35
OV121093.1	13	46.91	35.6
OV121094.1	14	45.71	35
OV121095.1	15	45.14	35
OV121096.1	16	44.64	35.1
OV121097.1	17	44.39	35.3
OV121098.1	18	35.3	35.5
OV121099.1	MT	0.02	31.1
-	unplaced	2.13	37

The estimated Quality Value (QV) of the final assembly is 56.5 with *k*-mer completeness of 99.99%, and the assembly has a BUSCO v5.3.2 completeness of 85.6% (single = 85.0, duplicated = 0.6%), using the mollusca_odb10 reference set ( *n* = 5,295).

Metadata for specimens, spectral estimates, sequencing runs, contaminants and pre-curation assembly statistics can be found at https://links.tol.sanger.ac.uk/species/1620919.

## Methods

### Sample acquisition and nucleic acid extraction

Two *Phorcus lineatus* specimens (xgPhoLine1 and xgPhoLine2) were collected from Godrevy in Cornwall, UK (latitude 50.23786, longitude –5.39607) on 22 June 2020. The specimens were collected by Nova Mieszkowska and Rob Mrowicki (Marine Biological Association) and identified by Nova Mieszkowska. They were collected by hand and placed in a sample bag, and then preserved in 100% ethanol.

DNA was extracted at the Tree of Life laboratory, Wellcome Sanger Institute (WSI). The xgPhoLine1 sample was weighed and dissected on dry ice with tissue set aside for Hi-C sequencing. Muscle tissue was cryogenically disrupted to a fine powder using a Covaris cryoPREP Automated Dry Pulveriser, receiving multiple impacts. Fragment size analysis of 0.01–0.5 ng of DNA was then performed using an Agilent FemtoPulse. High molecular weight (HMW) DNA was extracted using the Qiagen MagAttract HMW DNA extraction kit. Low molecular weight DNA was removed from a 200-ng aliquot of extracted DNA using 0.8X AMpure XP purification kit prior to 10X Chromium sequencing; a minimum of 50 ng DNA was submitted for 10X sequencing. HMW DNA was sheared into an average fragment size between 12–20 kb in a Megaruptor 3 system with speed setting 30. Sheared DNA was purified by solid-phase reversible immobilisation using AMPure PB beads with a 1.8X ratio of beads to sample to remove the shorter fragments and concentrate the DNA sample. The concentration of the sheared and purified DNA was assessed using a Nanodrop spectrophotometer and Qubit Fluorometer and Qubit dsDNA High Sensitivity Assay kit. Fragment size distribution was evaluated by running the sample on the FemtoPulse system.

RNA was extracted from muscle tissue of xgPhoLine2 in the Tree of Life Laboratory at the WSI using TRIzol, according to the manufacturer’s instructions. RNA was then eluted in 50 μl RNAse-free water and its concentration assessed using a Nanodrop spectrophotometer and Qubit Fluorometer using the Qubit RNA Broad-Range (BR) Assay kit. Analysis of the integrity of the RNA was done using Agilent RNA 6000 Pico Kit and Eukaryotic Total RNA assay.

### Sequencing

Pacific Biosciences HiFi circular consensus and 10X Genomics read cloud DNA sequencing libraries were constructed according to the manufacturers’ instructions. Poly(A) RNA-Seq libraries were constructed using the NEB Ultra II RNA Library Prep kit. DNA and RNA sequencing were performed by the Scientific Operations core at the WSI on Pacific Biosciences SEQUEL II (HiFi) and Illumina NovaSeq 6000 (RNA-Seq and 10X) instruments. Hi-C data were also generated from muscle tissue of xgPhoLine1 using the Arima2 kit and sequenced on the Illumina NovaSeq 6000 instrument.

### Genome assembly, curation and evaluation

Assembly was carried out with Hifiasm ( Cheng *et al.*, 2021) and haplotypic duplication was identified and removed with purge_dups ( Guan *et al.*, 2020). One round of polishing was performed by aligning 10X Genomics read data to the assembly with Long Ranger ALIGN, calling variants with FreeBayes ( Garrison & Marth, 2012). The assembly was then scaffolded with Hi-C data ( Rao *et al.*, 2014) using YaHS ( Zhou *et al.*, 2023). The assembly was checked for contamination and corrected using the gEVAL system ( Chow *et al.*, 2016) as described previously ( Howe *et al.*, 2021). Manual curation was performed using gEVAL, HiGlass ( Kerpedjiev *et al.*, 2018) and Pretext ( Harry, 2022). The mitochondrial genome was assembled using MitoHiFi ( Uliano-Silva *et al.*, 2022), which runs MitoFinder ( Allio *et al.*, 2020) or MITOS ( Bernt *et al.*, 2013) and uses these annotations to select the final mitochondrial contig and to ensure the general quality of the sequence.

A Hi-C map for the final assembly was produced using bwa-mem2 ( Vasimuddin *et al.*, 2019) in the Cooler file format ( Abdennur & Mirny, 2020). To assess the assembly metrics, the *k*-mer completeness and QV consensus quality values were calculated in Merqury ( Rhie *et al.*, 2020). This work was done using Nextflow ( Di Tommaso *et al.*, 2017) DSL2 pipelines “sanger-tol/readmapping” ( Surana *et al.*, 2023a) and “sanger-tol/genomenote” ( Surana *et al.*, 2023b). The genome was analysed within the BlobToolKit environment ( Challis *et al.*, 2020) and BUSCO scores ( Manni *et al.*, 2021; Simão *et al.*, 2015) were calculated.

Table 3 contains a list of relevant software tool versions and sources.

**Table 3.** Software tools: versions and sources.

Software tool	Version	Source
BlobToolKit	4.0.7	https://github.com/blobtoolkit/blobtoolkit
BUSCO	5.3.2	https://gitlab.com/ezlab/busco
FreeBayes	1.3.1-17-gaa2ace8	https://github.com/freebayes/freebayes
gEVAL	N/A	https://geval.org.uk/
Hifiasm	0.15.3	https://github.com/chhylp123/hifiasm
HiGlass	1.11.6	https://github.com/higlass/higlass
Long Ranger ALIGN	2.2.2	https://support.10xgenomics.com/genome-exome/ software/pipelines/latest/advanced/other-pipelines
Merqury	MerquryFK	https://github.com/thegenemyers/MERQURY.FK
MitoHiFi	2	https://github.com/marcelauliano/MitoHiFi
PretextView	0.2	https://github.com/wtsi-hpag/PretextView
purge_dups	1.2.3	https://github.com/dfguan/purge_dups
YaHS	1.0	https://github.com/c-zhou/yahs
sanger-tol/genomenote	v1.0	https://github.com/sanger-tol/genomenote
sanger-tol/readmapping	1.1.0	https://github.com/sanger-tol/readmapping/tree/1.1.0

### Legal and ethical review process for Darwin Tree of Life Partner submitted materials

The materials that have contributed to this genome note have been supplied by a Darwin Tree of Life Partner.

The submission of materials by a Darwin Tree of Life Partner is subject to the **‘Darwin Tree of Life Project Sampling Code of Practice’**, which can be found in full on the Darwin Tree of Life website here. By agreeing with and signing up to the Sampling Code of Practice, the Darwin Tree of Life Partner agrees they will meet the legal and ethical requirements and standards set out within this document in respect of all samples acquired for, and supplied to, the Darwin Tree of Life Project.

Further, the Wellcome Sanger Institute employs a process whereby due diligence is carried out proportionate to the nature of the materials themselves, and the circumstances under which they have been/are to be collected and provided for use. The purpose of this is to address and mitigate any potential legal and/or ethical implications of receipt and use of the materials as part of the research project, and to ensure that in doing so we align with best practice wherever possible.

The overarching areas of consideration are:

- Ethical review of provenance and sourcing of the material
- Legality of collection, transfer and use (national and international)

Each transfer of samples is further undertaken according to a Research Collaboration Agreement or Material Transfer Agreement entered into by the Darwin Tree of Life Partner, Genome Research Limited (operating as the Wellcome Sanger Institute), and in some circumstances other Darwin Tree of Life collaborators.

## Data Availability

European Nucleotide Archive: *Phorcus lineatus* (thick top shell). Accession number PRJEB48398; https://identifiers.org/ena.embl/PRJEB48398 ( Wellcome Sanger Institute, 2022). The genome sequence is released openly for reuse. The *Phorcus lineatus* genome sequencing initiative is part of the Darwin Tree of Life (DToL) project. All raw sequence data and the assembly have been deposited in INSDC databases. The genome will be annotated using available RNA-Seq data and presented through the Ensembl pipeline at the European Bioinformatics Institute. Raw data and assembly accession identifiers are reported in Table 1.

## Author information

Members of the Marine Biological Association Genome Acquisition Lab are listed here:

https://doi.org/10.5281/zenodo.4783604.

Members of the Darwin Tree of Life Barcoding collective are listed here: https://doi.org/10.5281/zenodo.4893703.

Members of the Wellcome Sanger Institute Tree of Life programme are listed here: https://doi.org/10.5281/zenodo.4783585.

Members of Wellcome Sanger Institute Scientific Operations: DNA Pipelines collective are listed here: https://doi.org/10.5281/zenodo.4790455.

Members of the Tree of Life Core Informatics collective are listed here: https://doi.org/10.5281/zenodo.5013541.

Members of the Darwin Tree of Life Consortium are listed here: https://doi.org/10.5281/zenodo.4783558.
