# Supplementary material for: Genome sequence for the thick topshell, Phorcus lineatus (da Costa, 1778)
Source: Wellcome Open Res. 2023 Jul 11;8:296. [Version 1] doi: 10.12688/wellcomeopenres.19485.1 (PMC10444997; doi:10.12688/wellcomeopenres.19485.1)
Supplement: Peer review history

# Reviewer report: Reviewer response for version 1

**DOI:** 10.21956/wellcomeopenres.21583.r64688

**Published:** 2023-09-26

**Authors:** Masa-aki Yoshida

**Affiliations:** 1 Oki Marine Biological Station, Faculty of Life and Environmental Sciences, Shimane University, Matsue, Japan

The paper by Mieszkowska and Mrowicki combines data from PacBio and 10x to represent the genome of *Phorcus lineatus* in good quality to meet the Darwin Tree of Life criteria. By the Darwin Tree of Life's standard for publishing genomes, this genome data was shown to make the data widely available to various researchers. The report is commendable for covering the necessary and sufficient content for a genome report.

 I have a slight concern about "manual curation" to keep reproducibility of the analysis. However, the other Darwin Tree of Life genome papers seem to have the same description and seem to be acceptable, so we hope that a detailed method paper will be published someday.

Are sufficient details of methods and materials provided to allow replication by others?

Partly

Is the rationale for creating the dataset(s) clearly described?

Yes

Are the datasets clearly presented in a useable and accessible format?

Yes

Are the protocols appropriate and is the work technically sound?

Yes

Reviewer Expertise:

evolutionary biology and genome biology

I confirm that I have read this submission and believe that I have an appropriate level of expertise to confirm that it is of an acceptable scientific standard.

# Reviewer report: Reviewer response for version 1

**DOI:** 10.21956/wellcomeopenres.21583.r64678

**Published:** 2023-08-22

**Authors:** Marco Gerdol

**Affiliations:** 1 Department of Life Sciences, University of Trieste, Trieste, Italy

Mieszkowska and colleagues report the genome assembly of the thick topshell Phorcus lineatus, and abundant gastropod mollusk in the coasts of North-Eastern Atlantic. Interestingly, this species is considered a fast-responder to climate change, as suggested by its quickly expanding area of distribution towards northern latitudes over the past few decades.

 The assembly was generated using gold-standard technologies, i.e. a combination of PacBio HiFi reads, Illumina 10X and Hi-C libraries. These data allowed the assembly of a highly complete and contiguous assembly, which satisfied all VGP benchmarks, with the exception of BUSCO metrics. Nevertheless, the present version of the Mollusca dataset in OrthoDB is notoriously inappropriate for quality assessment in Mollusca, most likely due to the small number of high quality genomes used for its generation, thereby leading to significant underestimates of the actual quality of newly assembled molluscan genomes. The authors might therefore want to switch to a more reliable dataset for this assessment, such as the Metazoa ODB reference.

 As in all other Darwin Tree of Life reports, the published genome currently lacks annotation, as clearly mentioned in the data availability statement.

 The authors briefly mentioned the presence of a single probable heterozygous inversion, but did not actually report any data about heterozygosity itself, which can be easily estimated from k-mer spectra analysis, even though k-mer representation in the assembly was very high.

 If this data is available, the authors could briefly mentioned whether the assembly size and inferred number of chromosomes are consistent with previous cytogenetic estimates.

Are sufficient details of methods and materials provided to allow replication by others?

Yes

Is the rationale for creating the dataset(s) clearly described?

Yes

Are the datasets clearly presented in a useable and accessible format?

Yes

Are the protocols appropriate and is the work technically sound?

Yes

Reviewer Expertise:

molluscan genomics

I confirm that I have read this submission and believe that I have an appropriate level of expertise to confirm that it is of an acceptable scientific standard.

# Reviewer report: Reviewer response for version 1

**DOI:** 10.21956/wellcomeopenres.21583.r64686

**Published:** 2023-08-22

**Authors:** Melody Clark

**Affiliations:** 1 Natural Environment Research Council, Cambridge, England, UK

This article describes the genome sequencing and assembly of the thick topshell, Phorcus lineatus via the Darwin Tree of Life programme. As would be expected of a genome produced by the Sanger Institute, it is produced using a very robust standard pipeline and is of the highest quality. The background to the species is well described. The genome sequence report is produced using standard reporting metrics and displayed via standard bioinformatics packages. This gastropod genome is a useful addition to the expanding repertoire of mollusc genomes.

Are sufficient details of methods and materials provided to allow replication by others?

Yes

Is the rationale for creating the dataset(s) clearly described?

Yes

Are the datasets clearly presented in a useable and accessible format?

Yes

Are the protocols appropriate and is the work technically sound?

Yes

Reviewer Expertise:

Marine invertebrate responses to climate change, mollusc transcriptomics and genomics

I confirm that I have read this submission and believe that I have an appropriate level of expertise to confirm that it is of an acceptable scientific standard.

# Reviewer report: Reviewer response for version 1

**DOI:** 10.21956/wellcomeopenres.21583.r63531

**Published:** 2023-08-11

**Authors:** Rafael Zardoya

**Affiliations:** 1 Departamento de Biodiversidad y Biología Evolutiva, Museo Nacional de Ciencias Naturales (MNCN-CSIC), Madrid, Spain

The paper reports the sequencing at the chromosome level of the thick topshell genome-. The natural history of the species is well introduced and standard methods of sequencing, assembling and scaffolding are well explained. I have only few minor comments:

- the term "prosobranch" is outdated as it is from an old taxonomic classification of gastropods and represent a non-monophyletic group. I suggest using "vetigastropod" instead.
- I think "in the south and west of the UK. It has a geographic distribution extending" can be deleted, as it is local compared to the whole distribution.
- The BUSCO value is relatively low. This is normal with many gastropods. Have the authors try using the metazoan odb, it sometimes increases the BUSCO value.
- I know this is a report and must be short but it would be nice mentioning and comparing briefly this genome with that of the closely related *Chrysomallon squamiferum*

Are sufficient details of methods and materials provided to allow replication by others?

Yes

Is the rationale for creating the dataset(s) clearly described?

Yes

Are the datasets clearly presented in a useable and accessible format?

Yes

Are the protocols appropriate and is the work technically sound?

Yes

Reviewer Expertise:

Gastropod phylogenomics and comparative genomics

I confirm that I have read this submission and believe that I have an appropriate level of expertise to confirm that it is of an acceptable scientific standard.
